# The epidemiology, antibiotic resistance and post-discharge course of peritonsillar abscesses in London, Ontario

**DOI:** 10.1186/1916-0216-42-5

**Published:** 2013-01-31

**Authors:** Leigh J Sowerby, Zafar Hussain, Murad Husein

**Affiliations:** 1 Department of Otolaryngology, Schulich School of Medicine and Dentistry, University of Western Ontario, London, Ontario, Canada; 2 Division of Microbiology, Department of Pathology, University of Western Ontario, London, Ontario, Canada; 3 Department of Otolaryngology, Division of Pediatric Otolaryngology, London Health Sciences Centre-Victoria Hospital, 800 Commissioners Road East, London, Ontario, N6A 5W9, Canada

**Keywords:** Epidemiology, Peritonsillar abscess, Antibiotic resistance, Microbiology, Pain, Post-operative follow-up

## Abstract

**Background:**

Peritonsillar abscesses (PTA) are a common complication of tonsillitis. Recent global epidemiological data regarding PTAs have demonstrated increasing antimicrobial resistance patterns. No similar studies have been conducted in Canada and no Canadian study has examined the post-discharge course of treated patients.

**Methods:**

A prospective observational study of the epidemiology, antibiotic resistance and post-discharge course of patients presenting with a peritonsillar abscess to the Emergency Department in London, Ontario over one year. A follow-up telephone survey was conducted 2–3 weeks after abscess drainage.

**Results:**

60 patients were diagnosed with an abscess, giving an incidence of 12/100,000. 46 patients were enrolled in the study; the average duration of symptoms prior to presentation was 6 days, with 51% treated with antibiotics prior to presentation. *Streptococcus pyogenes* and *Streptococcus anginosus* were present in 56% of isolates and of those, 7/23 (32%) of specimens demonstrated resistance to clindamycin. Eight patients were treated with clindamycin and had a culture that was resistant, yet only one had recurrence. Telephone follow-up was possible for 38 patients: 51% of patients reported a return to solid food within 2 days, and 75% reported no pain by 5 days. Resolution of trismus took a week or longer for 51%.

**Interpretation:**

Clindamycin resistance was identified in a third of *Streptococcus* isolates, which should be taken into account when prescribing antibiotics. Routine culture appears unnecessary as patients recover quickly from outpatient drainage and empiric therapy, with less pain than expected, but trismus takes time to resolve.

## Introduction

Peritonsillar abscesses (PTA) are the most common deep neck space infection and are a common complication of tonsillitis with potentially disastrous sequelae [1]. Epidemiological studies from around the world have reported an incidence of 10 to 37 per 100,000 people [1-3]. An untreated or improperly-treated peritonsillar infection can evolve into a parapharyngeal space abscess, or cause sepsis, airway obstruction, carotid pseudo-aneurysm and even death [1].

Very few papers have been published examining the Canadian perspective, with the most recent epidemiologic paper published in 1990 [4]. The treatment of this disease has changed significantly over time as the vast majority of patients are now treated with outpatient drainage. Several recent studies have demonstrated significant changes in both the microbiology of the bacteria and their resistance patterns to commonly used antibiotics [5,6].

The objectives of this study were two-fold. The first was to characterize the bacterial flora, epidemiology and antimicrobial resistance patterns of local peritonsillar abscesses. The second was to detail the post-discharge course of patients with drained peritonsillar abscesses, with regards to pain, oral intake, trismus and the need for pain medication, with the ultimate aim of optimizing outpatient management.

## Methods

After obtaining research ethics board approval, a prospective observational study was conducted in all four (three adult and one pediatric) Emergency Departments (ED) in London, Ontario, Canada from January 30^th^, 2009 to February 1^st^, 2010.

### Treatment/enrollment

**Figure 1:**
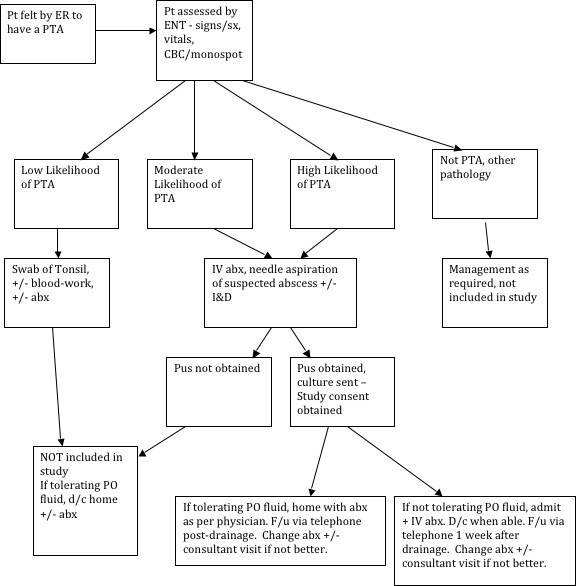
Treatment algorithm for peritonsillar abscesses.

All patients seen in consultation for a possible peritonsillar abscess were managed as per standard protocol, as detailed in Figure 1. All management of the case - including choice of antibiotic, admission, further investigations and follow-up - was left to the discretion of the treating physician. If pus was obtained, a specimen was sent for aerobic culture/sensitivity and the patient was asked to participate in the study. If they consented, a history/physical exam sheet was completed by the treating physician to record demographics and exam findings, and contact information was verified with the patient prior to discharge or admission.

### Post-discharge course

Follow-up for the patient was dictated by the treating physician. As part of the study, the patient was contacted 2 to 3 weeks post-drainage of their abscess for a telephone survey. The survey questionnaire asked about the number of days to solid food, days to full pain resolution, days requiring any pain medication, and days to resolution of trismus. If any signs of persistent disease were evident on questioning, follow-up was arranged for the patient. If bacteria with antibiotic resistance were cultured, the patient was informed, so as to help guide any future antibiotic therapy.

### Event capture

To ascertain the true participation rate, after completion of the study, the ED patient database was retrospectively reviewed using the *International Classification of Diseases, Ninth Revision* (ICD-9-CM) code for peritonsillar abscess to identify peritonsillar abscess as either a primary or secondary diagnosis. The medical records of all identified patients were then reviewed to assess whether or not a peritonsillar abscess had actually been diagnosed. The resulting list was then cross-referenced with enrolled patients.

### Statistical analysis

Descriptive statistical comparisons were calculated for demographic data. For inferential statistical comparisons, SPSS® version 13.0 was used. Chi-squared tests of independence were used for categorical variables, student’s t-test was used for continuous variables and ANOVA was used in the setting of multiple variables. All tests were two-tailed and p ≤ 0.05 was set as the threshold for statistical significance.

## Results

### Epidemiology

**Figure 2:**
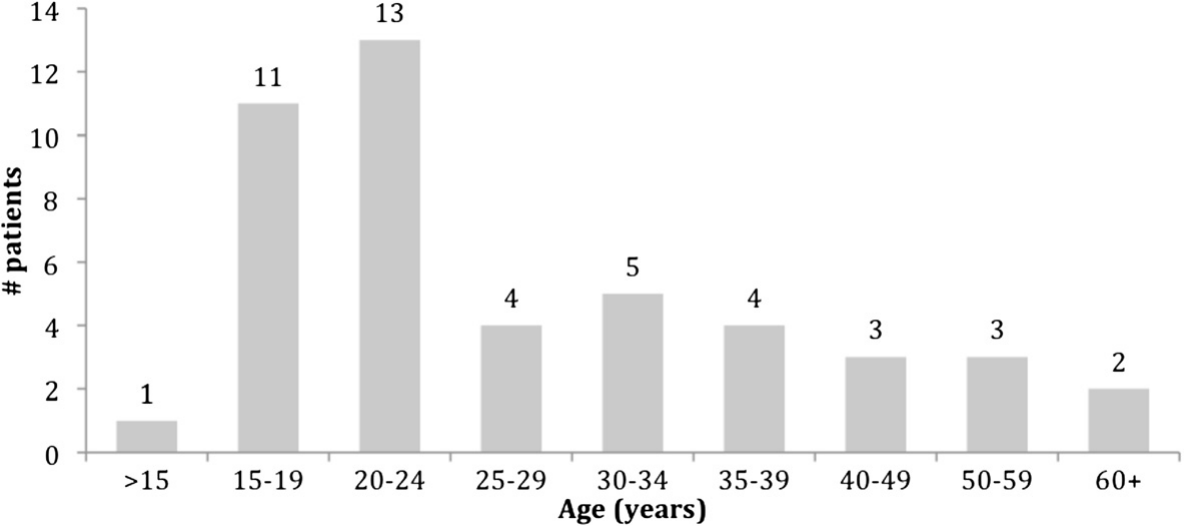
Age distribution of patients with peritonsillar abscesses.

**Figure 3:**
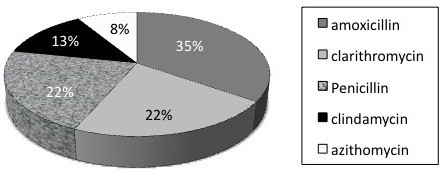
Prior antibiotic usage for tonsillitis/pharyngitis.

A total of 46 patients were enrolled in the study. Gender distribution was approximately equal, with 25 males (54%) enrolled. Figure 2 demonstrates the age distribution. Duration of symptoms prior to presentation averaged 6 days, with only 3 patients (6.7%) presenting within two days of symptom onset. Only 15% of patients admitted to smoking and half of the patients had taken antibiotics for pharyngitis over the preceding month. The antibiotic prescribed prior to presentation for pharyngitis/tonsillitis was wide ranging, as seen in Figure 3. Only 15 patients (24%) had a temperature of greater than 38°C at presentation but an elevated white blood cell count (greater than 11.0 ×10^9^/L) was present in 82% of patients, and 91% had an elevated neutrophil count (greater than 7.5 ×10^9^/L). Only two patients (4.5%) had a positive heterophile antibody test. On physical exam, the classic symptoms of uvular deviation (94%), trismus (94%) and muffled voice (91%) were consistently present.

The volume of pus drained was 5 mL or less in 32 patients (71%) with an overall mean and median of 4 (+/−1.1) mL drained; there was no statistically significant relationship between the amount of pus drained and fever, white blood cell count, duration of symptoms or neutrophil count.

### Microbiology

Only 7% of patients had no growth (see Table 1 for full culture results). Streptococcus, either alone or in combination with other bacteria, was cultured in 25 of 46 patients (56%), and in 25 of 29 (81%) cultures in which a specific pathogen was identified. *Streptococcus pyogenes* and *Streptococcus anginosus* accounted for the vast majority of Streptococcus cultures, with *S. anginosus* present in 12 cultures and *S. pyogenes* in 11 cultures out of 42 patients with a positive culture. *Streptococcus pyogenes* was the sole organism in 11 of 12 (91%) cultures where it was identified while only 3 of 12 (25%) cultures with *S. anginosus* had a single organism cultured (p=0.001). The Gram stain was indicative of possible anaerobes by the presence of multiple organisms, such as Gram positive/negative rods and Gram negative cocci, that failed to grow in aerobic culture in 20 (44%) specimens. A single organism was cultured in 16 of 46 (36%) patients whereas multiple organisms were present in 26 of 46 (58%) cultures. Antimicrobial resistance was found in 11 of 23 tested *Streptococcus* specimens (48%), with resistance to clindamycin in 32%, and to erythromycin in 41%. All *Streptococcus* species were universally susceptible to penicillin. In the 5 *Staphylococcus* cultures, 3 (60%) were resistant to clindamycin and 4 (80%) to erythromycin. *S. aureus* was universally sensitive to oxacillin, vancomycin and sulphamethoxazole.

**Table 1:** Culture results from aspirated pus

**Bacteria type**	**N**	**%**
No growth	3	6.7%
Normal respiratory flora	11	24.4%
Group A Strep	11	24.4%
Strep Anginosus	9	20.0%
Other Strep	2	4.4%
Strep & Staph	3	6.7%
Staphylococcus alone	2	4.4%
Anaerobe(s)	3	6.7%
Other mixed flora	1	2.2%
Data missing	1	2.2%
	46	100%

There was a tendency for those patients not on an antibiotic at the time of presentation to culture positive for *S. pyogenes* with 8 of 22 patients (36%) not on antibiotics positive for *S. pyogenes* versus 3 of 23 patients (13%) already on an antibiotic, but this was not statistically significant (p = 0.07). No such trend existed for *Streptococcus* overall (p = 0.42). Similarly, there was a trend towards those not on an antibiotic to be febrile at presentation (7 of 19, 36.8%) relative to those already taking one (3 of 21, 14.3%), but this again failed to achieve statistical significance, whether tested by χ2 analysis (p = 0.10) or likelihood ratio (p = 0.097).

### Immediate management

All patients were treated with needle aspiration followed by a cruciate incision and drainage of any residual pus. A total of 7 patients (15%) were admitted for intravenous hydration, therapy and observation. The most common reason for admission was intolerance of oral intake (5 patients), followed by observation for potential airway concerns (2 patients). Duration of admission was 2 days or less for 5 of the 7 inpatients (71%). Of the patients discharged home from the ED, 5 patients (13%) returned to the Emergency Room for reassessment but only 2 of those patients had re-accumulation of pus. Re-drainage of the abscess was required in a total of 5 patients (11%), 3 of whom were still admitted when re-drained. In fact, hospitalization was clearly associated with pus re-accumulation (p=0.003). Re-accumulation of pus tended to be associated with higher initial pus drainage volumes, with the difference approaching statistical significance (p=0.082). Recurrence (abscess formation greater than 1 month after initial drainage) during the study period occurred in 1 patient, who had also had re-accumulation after initial drainage. None of the 5 patients with recurrence or re-accumulation were smokers.

**Figure 4:**
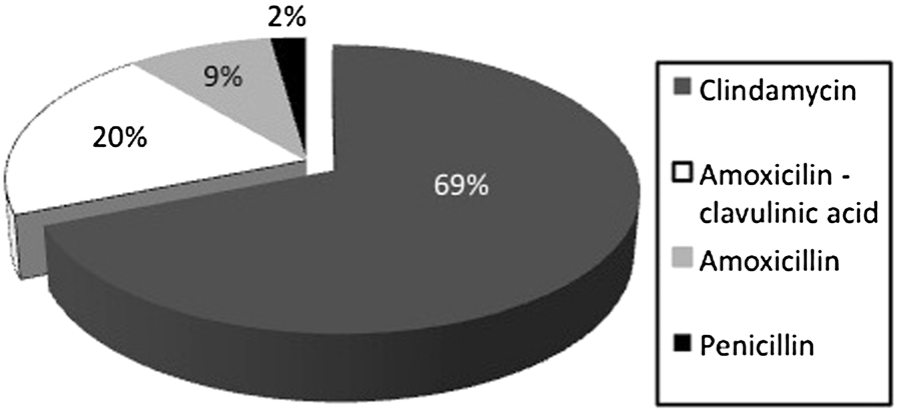
Choice of antibiotic post-drainage of abscess.

Patients were predominantly prescribed clindamycin (31 patients, 69%); Figure 4 details the remaining antibiotic choices. Of the patients with re-accumulation of pus, one grew *Haemophilus influenzae*, one grew *Fusobacterium necrophorum*, two had cultures with normal respiratory flora, and the last grew *S. pyogenes.* All 5 patients had been treated initially with clindamycin, and were switched to a different broad-spectrum antibiotic after re-drainage. Sensitivity results were available for two of the five patients; the cultured *S. pyogenes* was resistant to both erythromycin and clindamycin, while *F. necrophorum* was sensitive to clindamycin, metronidazole and penicillin.

### Post-discharge course

Of all the patients sent home on clindamycin, 8 of 31 (26%) had cultures return resistant to it and one patient did not take any antibiotic after drainage secondary to financial concerns. One of these patients was changed to a different antibiotic after presenting with recurrence, but all 8 other patients had resolution of their symptoms and were not given a different medication. All patients with cultured resistance were informed of such and were told what would be an appropriate antibiotic choice should their symptoms recur. No patients developed *Clostridium difficile* colitis.

**Figure 5:**
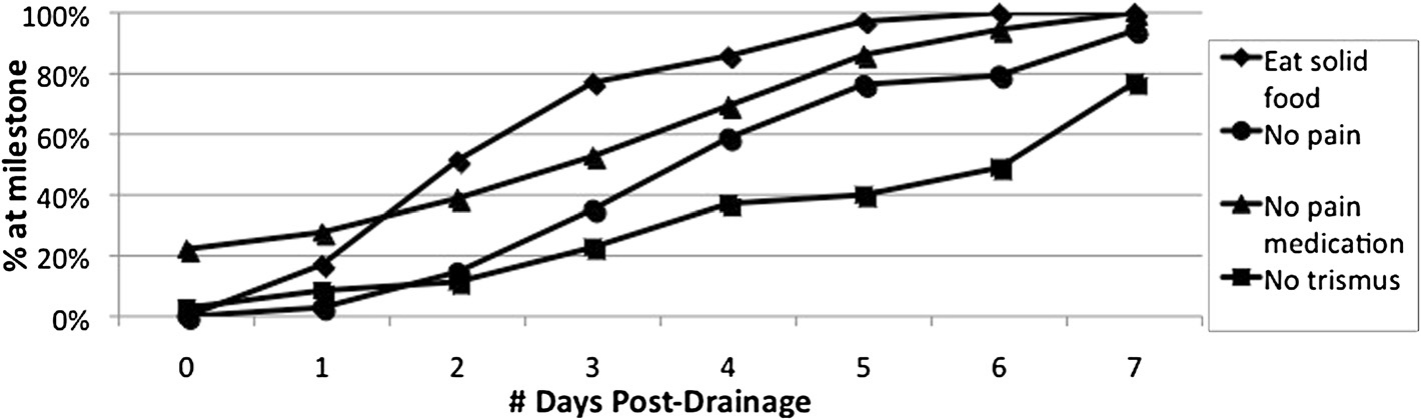
Number of days to resolution of symptoms.

A total of 38 patients (83%) were able to be contacted via telephone two to three weeks after abscess drainage and agreed to complete a short telephone questionnaire. Figure 5 presents all data for the 4 investigated endpoints. Half of patients (51%) were able to eat solid food within 2 days and most patients (75%) had complete resolution of any pain by 5 days. Pain medication was not required by 22% of patients after discharge, and 70% did not require any pain medication after 3 days. Resolution of trismus generally took much longer, with 51% of patients still experiencing trismus at 7 days and 18% at 2 weeks.

### Event capture

A total of 108 patients presenting to the emergency room during the study period had their primary or secondary diagnosis coded as a peritonsillar abscess. A review of these charts identified all of the 46 study patients and an additional 14 patients that had a peritonsillar abscess but were not enrolled in the study, giving an enrollment rate of 77%. The population of the city of London in 2009 was estimated to be 362,000 people [7]; and, as 45 of the 60 identified patients were from the city of London proper (based upon the first three digits of their postal code), the annual incidence of confirmed peritonsillar abscesses was estimated as 12.4 per 100,000. There was no significant month-to-month or seasonal variation noted when date of presentation was examined.

## Discussion

This study characterized several important facets of the epidemiology and management of peritonsillar abscesses. The most commonly affected demographic consists of those in their late-teens to early 30s; and virtually all had the classic symptoms of trismus, muffled voice and uvular deviation. Interestingly, fever was only found in 25% of our study participants, but the vast majority (91%) of patients had an elevated neutrophil count on presentation. Bacterial cultures found Streptococcus species in just over half of all specimens; all except for 2 cultures yielded some growth. It was surprising to discover that 32% of Streptococcus isolates and 60% of Staphylococcus isolates were resistant to clindamycin but that the use of culture results rarely impacted clinical decision-making. Most importantly, however, was that of the 9 patients treated with an ineffective antibiotic, 8 had complete resolution. It is possible that the simple act of draining the abscess is effective treatment and that antibiotic therapy may not be necessary. A German study investigated this prospectively in abscesses treated with tonsillectomy and found no difference between the treatment and control groups [8]. Generalizing these findings to needle drainage is likely not valid, but does provide further evidence that definitive drainage of the abscess may well be sufficient. Further study to investigate this possibility is warranted but would be challenging to undertake.

The vast majority of patients were able to be treated as outpatients, with 15% admitted for further therapy and observation. It is very interesting to note that 60% of the re-accumulations were in those patients admitted. The persistence of symptoms after drainage should thus alert the clinician to the high likelihood of re-accumulation or residual pus. In our patients, pain over the course of recovery was much less than anticipated; and perhaps the most surprising finding was the low use of pain medication. The standard discharge prescription at our centre consists of 30 doses of narcotic analgesia (acetominophen with codeine). Given that 22% of our patients did not use any pain medication at all, and that 70% did not require any pain medication beyond 3 days, a significant amount of narcotic is being dispensed and not used.

One important difference in the epidemiology from previous studies was that the 15% incidence of smoking was actually lower than the 17.3% incidence for London-Middlesex county reported in 2009 [9]. This is contrary to a previous Canadian study that found smoking to be significantly increased in patients with abscesses [10]. The prevalence of anaerobes in peritonsillar abscesses is unknown in Canada but it can be reasonably inferred from Gram stains that our rate of anaerobic involvement was 44%. This could be important, as a trend towards increasing anaerobic growth rates has been identified in Israel, increasing from 6.8% to 37% over three years and anaerobic presence has been shown to be a risk factor for recurrence [11,12]. Both the number of patients requiring hospitalization and the number of patients requiring re-aspiration was consistent with what has been reported previously in the literature, with past numbers for admission ranging from 3–14% and from 4–16% for re-drainage [1,13,14].

Generally, the current empirical choice of antibiotic at our centre has been clindamycin due to its broad Gram-positive and anaerobic coverage. Both clindamycin and penicillin with metronidazole are suggested by the American Academy of Otolaryngology as appropriate initial intravenous therapy [15]. For outpatient therapy, it has been suggested that either oral clindamycin, penicillin and metronidazole or amoxicillin-clavulinic acid are appropriate [16,17]. Megalamani *et al*. reported an increasing trend towards penicillin resistance in Gram-positive isolates from peritonsillar abscesses [5]; but, in our microbiology lab, Streptococcus specimens have remained almost universally susceptible to penicillin. The results of a different retrospective study revealed a significant increase in both the resistance to clindamycin in isolates from PTA cultures and the presence of anaerobic bacteria [6]. This is likely secondary to indiscriminate use of antibiotics, incomplete courses of antibiotic therapy and the use of second-line antibiotics when not clinically indicated. Based on the findings in our study, and other recent literature reports, clindamycin should be reserved as a second-line antibiotic with either penicillin and metronidazole or amoxicillin-clavulinic acid being prescribed as first-line therapy. Use of amoxicillin, however, would not be recommended in patients with concurrent infectious mononucleosis [17]. Lastly, as previously reported in three papers, culture results rarely affected clinical decision-making, calling into question the cost-effectiveness of routine culture of PTAs and suggesting that it should be reserved only for specific cases [17-20].

Several limitations were encountered in our study. Patients were contacted two to three weeks after drainage, introducing the possibility of recall bias. To mitigate this, patients were instructed that follow-up would be occurring and every attempt was made to ensure that follow-up occurred by 3 weeks post-drainage at the latest, with those unable to be reached in that time period excluded from the follow-up portion of the study. It is possible that our failure to enroll certain otherwise-eligible subjects in the study was because of unique demographics, like extremes in age or complications. On reviewing these data, however, this was not found to be the case. Further confounding factors could be the experience of the treating physician (as most are treated by junior and intermediate resident physicians), the lack of anaerobic culture (due to budget constraints) and variations in individual treatment techniques. However, by examining this population without intervening in the treatment protocol adds a more accurate picture of the outcome of all patients treated for peritonsillar abscesses in London. Lastly, the statistical comparisons between microbial groups were underpowered and may well be valid conclusions. Further study would help investigate this further.

## Conclusions

The epidemiological findings in this study are consistent with what has been described previously in the literature, the exception being that smoking was not increased in our study group. The majority of drained abscesses grew a Streptococcus species and a surprisingly high 32% rate of resistance to clindamycin was discovered. That said, antibiotic therapy was typically empiric and was by-and-large not altered, appearing to render the routine culturing of specimens unnecessary. Abscesses tended to resolve despite being treated with an antibiotic against which the pathogen was culture-proven resistant, and patients required much less pain medication than what traditionally is prescribed.

## Competing interests

The authors have no financial interests to declare.

## Authors’ contributions

LS conceptualized the study idea, enrolled patients, analyzed the data and wrote the draft manuscript. MH participated in study design, data analysis and edited the draft manuscript extensively. ZH performed microbiological analysis, study design and manuscript editing. All authors read and approved the manuscript.

## Confirmation of original material

This material has never been published and is not currently under evaluation for any other peer-reviewed publication.

## Meeting presentation

Presented at the 65th Annual Canadian Society of Otolaryngology – Head and Neck Surgery meeting in Victoria, British Columbia, Canada - May 2011.
